# Monte Carlo calculated absorbed‐dose energy dependence of EBT3 and EBT4 films for 5–200 MeV electrons and 100 keV–15 MeV photons

**DOI:** 10.1002/acm2.14529

**Published:** 2024-09-13

**Authors:** Nathan Clements, Magdalena Bazalova‐Carter

**Affiliations:** ^1^ Department of Physics and Astronomy University of Victoria Victoria British Columbia Canada

**Keywords:** dosimetry, EBT3, EBT4, Monte Carlo simulation, radiochromic film

## Abstract

**Purpose:**

To use Monte Carlo simulations to study the absorbed‐dose energy dependence of GAFChromic EBT3 and EBT4 films for 5–200 MeV electron beams and 100 keV–15 MeV photon beams considering two film compositions: a previous EBT3 composition (Bekerat et al.) and the final composition of EBT3/current composition of EBT4 (Palmer et al.).

**Methods:**

A water phantom was simulated with films at 5–50 mm depth in 5 mm intervals. The water phantom was irradiated with flat, monoenergetic 5–200 MeV electron beams and 100 and 150 keV kilovoltage and 1–15 MeV megavoltage photon beams and the dose to the active layer of the films was scored. Simulations were rerun with the films defined as water to compare the absorbed‐dose response of film to water, $f^{-1}\left(Q\right)=\frac{D_{film}}{D_{water}}$.

**Results:**

For electrons, the Bekerat et al. composition had variations in $f^{-1}\left(Q\right)$ of up to (1.9±0.1)% from 5 to 200 MeV. Similarly, the Palmer et al. composition had differences in $f^{-1}\left(Q\right)$ up to (2.5±0.2)% from 5 to 200 MeV. For photons, $f^{-1}\left(Q\right)$ varied up to (2.4±0.3)% and (4.5±0.7)% from 100 keV to 15 MeV for the Bekerat et al. and Palmer et al. compositions, respectively. The depth of films did not appear to significantly affect $f^{-1}\left(Q\right)$ for photons at any energy and for electrons at energies > 50 MeV. However, for 5 and 10 MeV electrons, decreases of up to (10.2±1.1)% in $f^{-1}\left(Q\right)$ were seen due to stacked films and increased beam attenuation in films compared to water.

**Conclusions:**

The up to (2.5±0.2)% and (4.5±0.7)% variations in $f^{-1}\left(Q\right)$ for electrons and photons, respectively, across the energies considered in this study indicate the importance of calibrating films with the energy intended for measurement. Additionally, this work emphasizes potential issues with stacking films to measure depth dose curves, particularly for electron beams with energies ≤10 MeV.

## 1 INTRODUCTION

Radiochromic films are highly utilized dosimeters clinically and in research in both x‐ray imaging^1^, ^2^, ^3^ ^]^ and radiation therapy.^4^, ^5^, ^6^ Radiochromic films, such as GAFChromic EBT3 and EBT4 (Ashland Inc, Bridgewater, NJ, USA), benefit from a high spatial resolution (∼25μm), near‐tissue equivalence, a dynamic dose range (0.1–20 Gy), and not requiring post‐exposure treatment.^7^, ^8^ GAFChromic films have been used with a wide variety of sources including monochromatic 25–35 keV synchrotron x‐ray beams,^9^ polychromatic 20–220 kVp beams generated by x‐ray tubes,^10^, ^11^ 6–25 MV photon beams and,^12^, ^13^, ^14^ 4–15 MeV electron beams^15^, ^16^ generated by clinical linear accelerators (linacs), 63–230 MeV proton beams,^17^, ^18^ 50–200 MeV very‐high energy electron (VHEE) beams,^19^, ^20^, ^21^, ^22^ and many more.

In this study, the absorbed‐dose energy dependence of EBT3 and EBT4 GAFChromic films is investigated. As described by Sutherland^23^ and Rogers,^24^ in general, the absorbed‐dose to the reference medium sensitivity of a detector, $S_{AD,med}$ (or the overall energy dependence of a detector) is given by the ratio of a detector reading $M_{det}$ to the true dose to medium value in the absence of the detector $D_{med}$:

(1) $$S_{AD,med}\left(D,\dot{D},Q,\theta ,\phi \right)=\frac{M_{det}\left(D,\dot{D},Q,\theta ,\phi \right)}{D_{med}\left(Q\right)}$$

where D is dose, $\dot{D}$ is dose rate, Q is beam quality, and θ and ϕ are geometric factors (film orientation in the case of film dosimetry).

In the case of constant dose, dose rate, and geometry, the absorbed‐dose sensitivity becomes the reciprocal of the total energy dependence:

(2) $$S_{AD,med}\left(Q\right)=\frac{1}{f\left(Q\right)\cdot k_{bq}\left(Q\right)}$$

where f(Q) is the absorbed‐dose energy dependence (or the extrinsic energy dependence) and $k_{bq}\left(Q\right)$ is the intrinsic energy dependence which together make up the total energy dependence.

For film dosimetry with water as the reference medium, the extrinsic energy dependence of the film is the ratio of dose to water to dose to film:

(3) $$f\left(Q\right)=\frac{D_{water}\left(Q\right)}{D_{film}\left(Q\right)}$$

The intrinsic energy dependence of the film is a measure of polymerization efficiency in the active material (color change) as a result of incident ionizing radiation and it is given by:

(4) $$k_{bq}\left(Q\right)=\frac{D_{film}\left(Q\right)}{netOD(Q)}$$

where netOD(Q) is the net optical density of the film post‐irradiation.

Monte Carlo (MC) simulations may be used to calculate the energy dependence of film (the extrinsic energy dependence), f(Q), by calculating the dose to film and dose to water. Previous MC simulation studies on the absorbed‐dose energy dependence for monoenergetic photon beams found that f(Q) varied within ±0.6% from 100 keV to 18 MeV for GAFChromic EBT and EBT2 films^23^ and by 2.3% for EBT3 between 10 keV and 18 MeV.^25^ A 2016 study by Sipilä et al. on the absorbed‐dose energy dependence of EBT3 films for 6–16 MeV electron beams found low energy dependence of ≤0.5%±1.6%.^26^ Considering protons, Shi et al. used MC simulations of EBT3 and EBT‐XD films to evaluate the continuous slowing down approximation (CSDA) range of protons for proton therapy applications and found good agreement between simulations and experiments.^27^

The total energy dependence of EBT3 film has been shown to be minimal for clinical 6–20 MV photon beams, while proving to have a significantly greater energy dependence for kilovoltage photon beams, particularly at lower energies of up to 20% for a 70 kV beam calibrated with a 6 MV beam.^28^, ^29^ To our knowledge no study on film energy dependence for high energy electrons of ≥ 50 MeV currently exists. One experimental VHEE study found that doses for 135 and 165 MeV electron beams measured with EBT2 films were within 3.5%–5.4% from MC simulations when a film calibration of a 20 MeV electron beam was used.^19^

The primary goal of this work was to simulate the dose‐response of EBT3 and EBT4 films using MC and examine the effects of film composition, beam energy, and particle type on dose‐to‐film compared to dose‐to‐water. A common use of GAFChromic films in radiotherapy is to measure the percentage depth dose (PDD) of a treatment beam. This is often done by sandwiching films at various depths between blocks of solid water.^22^, ^30^, ^31^ This study also investigates whether beam attenuation due to films upstream affects the measured dose of films at greater depths. Two EBT3 film compositions from previous studies were considered in this study. The first composition was an older and discontinued composition from a 2014 study by Bekerat et al.^32^ The second composition was considered as defined by Palmer et al. in 2015^33^ which corresponded to the final composition of EBT3 and the current composition of EBT4. This composition was also stated as the EBT3 composition in the American Association of Physicists in Medicine (AAPM) Task Group Report 235 on radiochromic film dosimetry.^4^ The most notable differences between the two compositions are the presence of aluminum and the lack of sulfur, chlorine, and bromine in the Palmer et al. composition. Though it is a past composition, the Bekerat et al. composition was considered to highlight that different batches/compositions of the same film model can be significantly different in their response to dose.

## 2 METHODS

### 2.1 Simulation software

TOol for PArticle Simulation (TOPAS) v3.9^34^, ^35^ was used to run all MC simulations. For photon beam simulations, the physics module used was the “g4em‐standard‐opt4” package and for the electron beam simulations, the physics list comprised the “g4em‐standard_opt4”, “g4decay”, “g4h‐phy_FTFP_BERT_HP”, and “g4em‐lowep” modules which have been used to cover the physics of very‐high‐energy‐electrons. The additional physics modules were chosen based on previous very‐high‐energy electron studies using TOPAS which found low (∼3%) discrepancy between film measurements and MC simulations.^21^ A cutoff distance of 5μm for all particles was applied for all simulations, which is one‐fifth of the active layer of EBT3 and EBT4 films. All other physics parameters were kept default. 40M histories were run for the electron beam simulations, 900M histories were run for the photon beams ≤1 MeV, and 200M histories were run for photon beams >1 MeV. Simulations were run on the Digital Research Alliance of Canada's NARVAL computer cluster using 64 CPUs, run time was between 30 mins and 5 h.

### 2.2 MC simulations of EBT3 and EBT4 films in a water phantom

**FIGURE 1:**
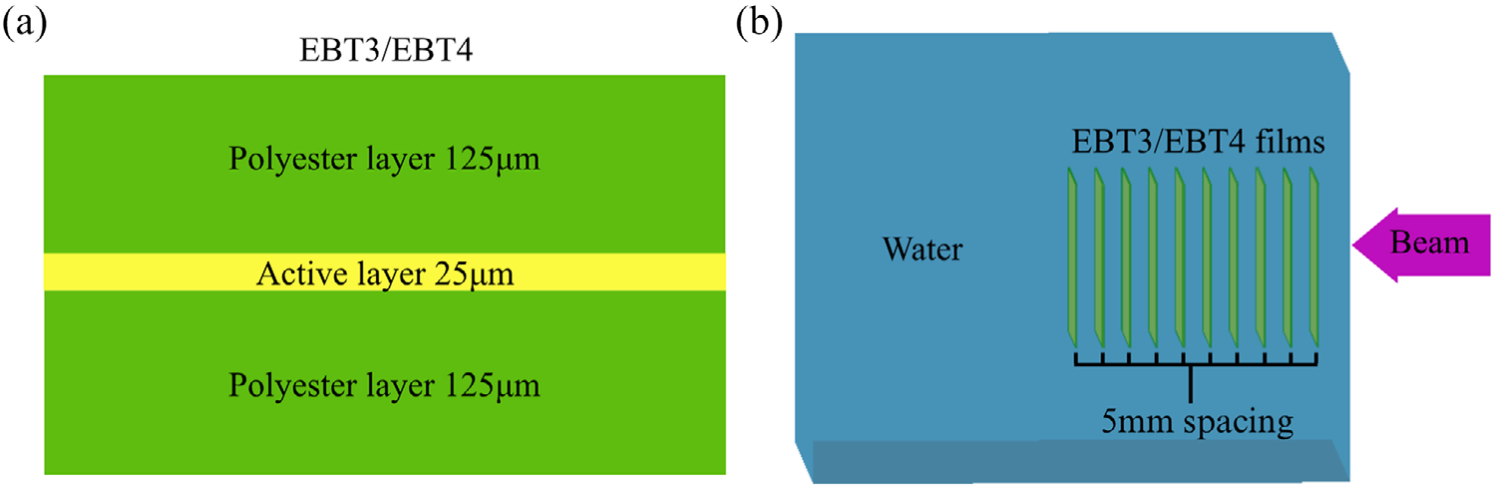
(a) EBT3/EBT4 film schematic with polyester base layers and the active layer indicated. (b) The water phantom simulation setup in TOPAS MC software. MC, Monte Carlo.

Films were placed in a water phantom at depths of 5–50 mm in 5 mm intervals, as shown in Figure 1b. The beam was defined as a flat, monoenergetic, rectangular 2 cm × 2 cm beam placed at a 1 cm distance from the water phantom in all simulations. Electrons with energies of 5, 10, 50, 100, and 200 MeV and photons with energies of 100 and 150 keV and 1, 2.5, 5, 10, 12.5, and 15 MeV were simulated.

Per manufacturer specifications, EBT3 and EBT4 films were defined as a 25μm active layer in between two 125μm polyester base layers (see Figure 1a). Two compositions for the active and base layers were considered in this study as presented by Palmer et al.^33^ and Bekerat et al.,^32^ and the mass fractions of each constituent for both film compositions are given in Table 1. Note that the change and advantage from EBT3 to EBT4 was in the packing of active materials in the fluid producing less noise^1^ and that the composition of EBT4 is the same as the final version of its predecessor EBT3, so the Palmer et al. composition should be accurate for EBT4 radiochromic films.^8^

**TABLE 1:** Thickness, density, and mass percent composition of EBT3 films.^32^, ^33^.

			Mass composition (%)									
Material	Thickness (μm)	Density ($\frac{g}{{\mathrm{cm}}^{3}}$)	H	Li	C	N	O	Na	Al	S	Cl	Br
EBT3 ‐ Palmer et al.												
Polyester base	125	1.35	4.2		62.5		33.3					
Active layer	25	1.2	8.8	0.6	51.1		32.8		6.7			
EBT3 ‐ Bekerat et al.												
Polyester base	125	1.35	4.0		63.0		33.0					
Active layer	25	1.2	9.7	0.9	58.4	0.1	28.4	0.4		0.2	1.1	0.8

The mean dose to medium and the standard deviation were scored in the active layer of the simulated films for both film compositions. All films were simulated and had dose scored simultaneously so that the effect of film stacking could also be studied. The simulations were then repeated with all film layers defined as water to compare dose‐response on film to dose to water. Primarily the first film was used to analyze the absorbed‐dose energy dependence without the influence of film stacking.

### 2.3 Film response analysis

To study the extrinsic dose‐response of the simulated EBT3 and EBT4 films, $f^{-1}\left(Q\right)$ was calculated as $D_{film}/D_{water}$ (as in Equation 1) for all beams at all film depths. Uncertainty was calculated as a combination of the statistical uncertainty of 40 mm × 40 mm regions of interest centered on the beam and MC uncertainty calculated in TOPAS.

## 3 RESULTS

**FIGURE 2:**
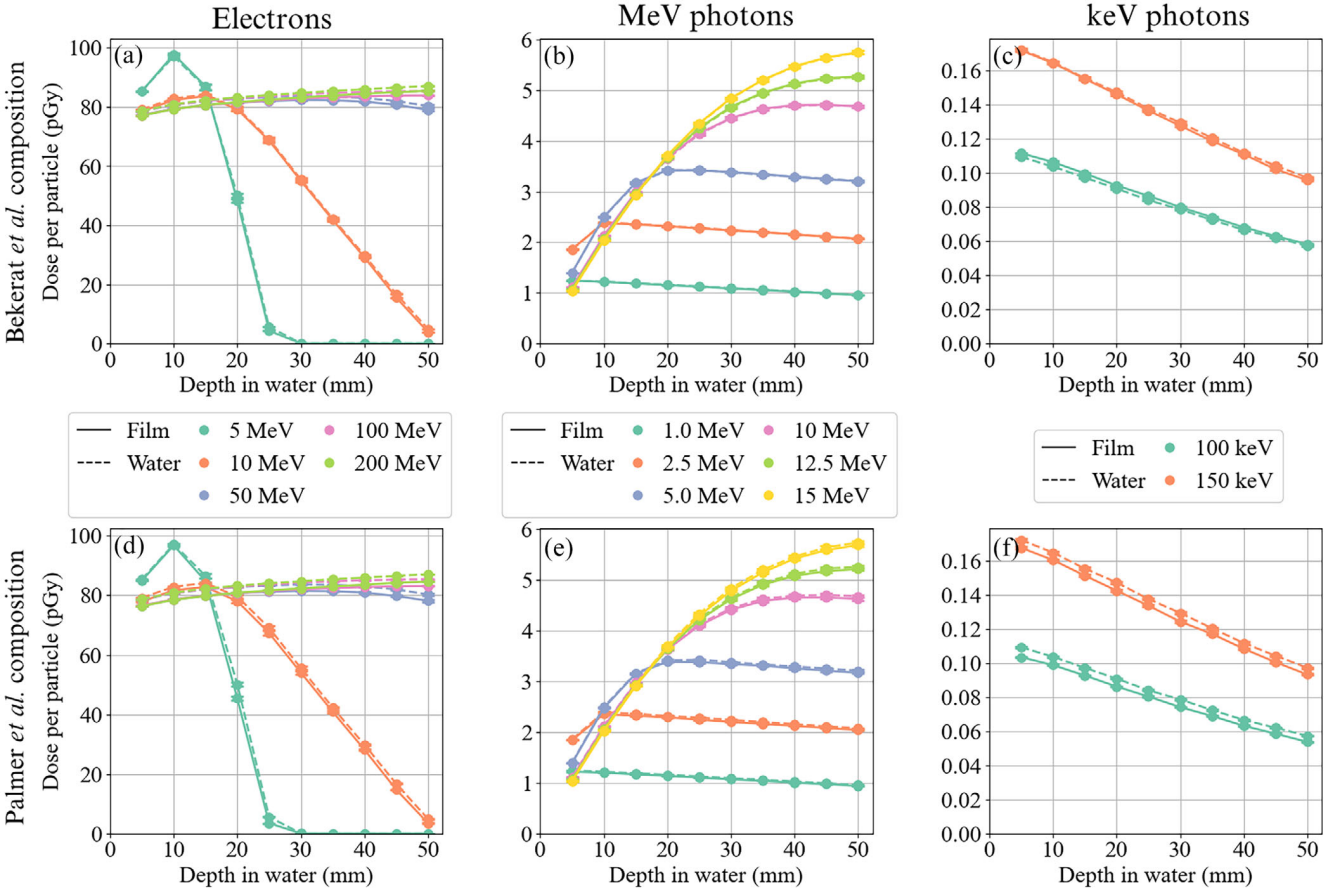
Dose per particle to film and water with depth in water for the Bekerat et al. (a–c) and Palmer et al. (d–f) film compositions for 5, 10, 50, 100, and 200 MeV electrons (a,d), 1, 2.5, 5, 10, 12.5, and 15 MeV photons (b,e), and 100 and 150 keV photons (c,f).

Dose‐to‐film, $D_{film}$, and dose‐to‐water, $D_{water}$, plots as a function of depth in water are shown in Figure 2 for 5–200 MeV electrons and 100 keV–15 MeV photon beams.

**FIGURE 3:**
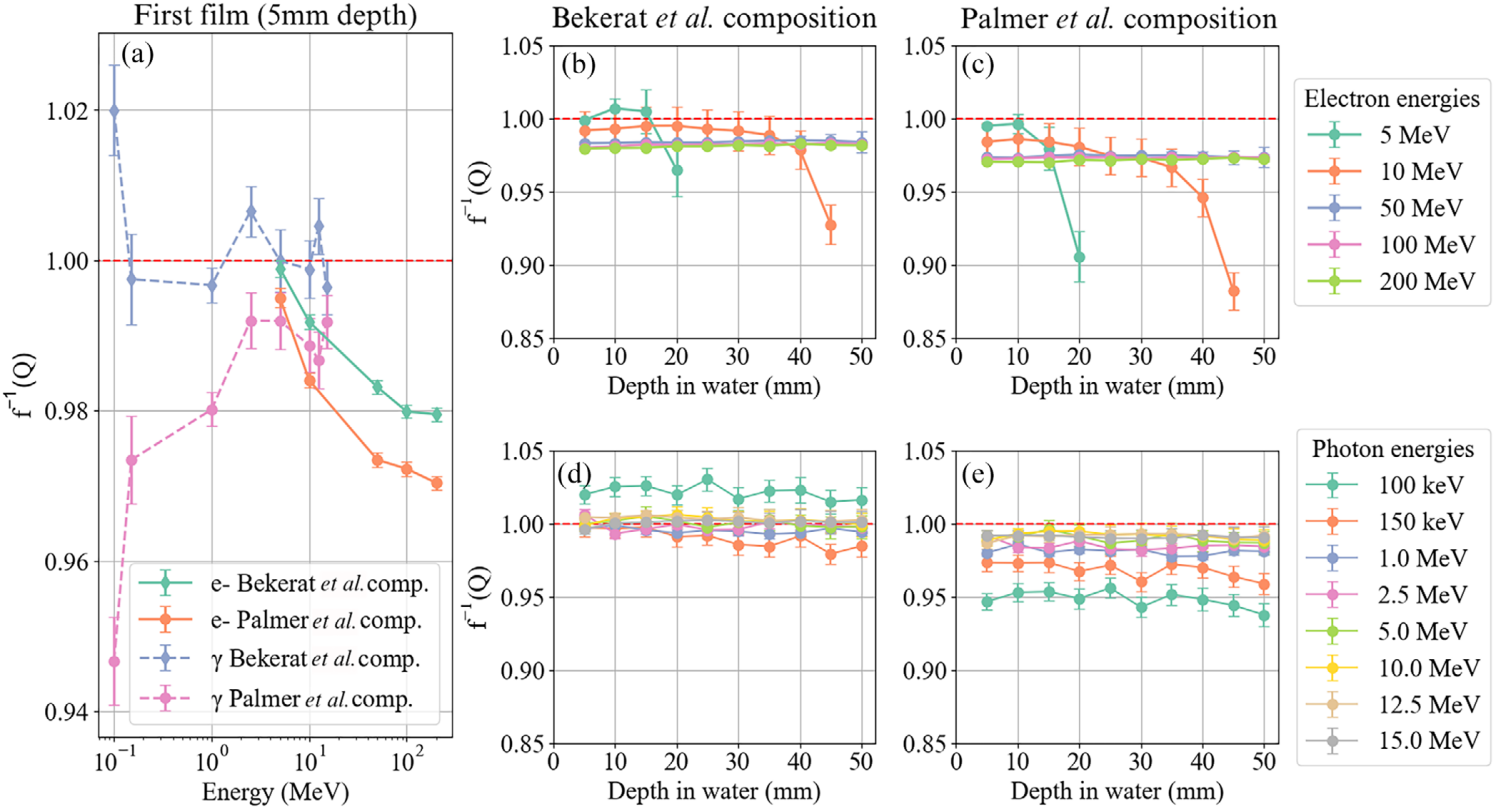
(a) Effects of energy on $f^{-1}\left(Q\right)$ for the first film at 5 mm depth for 5–200 MeV electron beams and 100 keV–15 MeV photon beams and both the Bekerat et al. and Palmer et al. film compositions. (b–e) $f^{-1}\left(Q\right)$ with depth in water for (b,d) the Bekerat et al. film composition and (c,e) the Palmer et al. film composition and for (b,c) 5–200 MeV electrons and (d,e) 100 keV–15 MeV photons. In each graph, a dashed red line indicates unity.

$f^{-1}\left(Q\right)$ was examined as a function of energy (Figure 3a) for the film placed at 5‐mm depth and as a function of depth for the Bekerat et al. and Palmer et al. compositions (Figure 3b–e).

All electron beams at 5 mm depth showed film under‐responded compared to water. For the clinical electron beams, the Bekerat et al. composition under‐responded by (0.1±0.1)% and (0.8±0.1)% at 5 mm depth for 5 and 10 MeV, respectively. Similarly, the Palmer et al. composition under‐responded by (0.5±0.1)% and (1.6±0.1)% at 5 mm depth for 5 and 10 MeV. At depths slightly shallower than their respective CSDA ranges (considering 20 mm for 5 MeV and 45 mm for 10 MeV^36^), the film under‐responses were (3.4±1.8)% and (6.4±1.2)% for 5 and 10 MeV, respectively, for the Bekerat et al. composition and (8.9±1.7)% and (10.2±1.1)% for the Palmer et al. composition. The high‐energy electrons (≥50 MeV) had greater under‐responses than the clinical energy electron beams, with an average of (2.1±0.1)% and (2.8±0.1)% at 5 mm depth for the Bekerat et al. and Palmer et al. compositions, respectively. These discrepancies varied minimally with depth, as seen in Figure 3b,c. The Bekerat et al. composition showed no discrepancy greater than 1.0% between film and water at any depth for any MeV photon beam and had an average discrepancy of (0.3±0.5)% across all MeV photon energies and depths. The Palmer et al. composition had an under‐response of (1.1±0.5)% averaged across depth and energies. For the 100 keV photon beam, the Bekerat et al. composition had an average film over‐response of (2.2±0.7)% across all depths of films and the Palmer et al. composition had an average under‐response of (5.2±0.7)% across all depths. For 150 keV photons, the average film under‐responses were (1.0±0.7)% and (3.1±0.7)% for the Bekerat et al. and Palmer et al. compositions, respectively. In additional simulations, $f^{-1}\left(Q\right)$ was found to vary by less than 1% up to depths of 20 cm for 50, 100, and 200 MeV electron beams and 10, 12.5, and 15 MeV photon beams.

In Figure 3a, $f^{-1}\left(Q\right)$ decreased for the electron beams with both film compositions in a very similar trend but the Palmer et al. composition consistently had a ∼0.8% greater under‐response than the Bekerat et al. composition. For the Bekerat composition, $f^{-1}\left(Q\right)$ decreased from 0.999±0.001 to 0.980±0.001 from 5 to 200 MeV and from 0.995±0.001 to 0.970±0.001 for the Palmer et al. composition. Specifically, these changes corresponded to variations in $f^{-1}\left(Q\right)$ of (1.9±0.1)% from 5 MeV electrons to 200 MeV electrons for the Bekerat et al. composition and (2.5±0.2)% for the Palmer et al. composition. For photons, the Bekerat et al. composition demonstrated a decreasing $f^{-1}\left(Q\right)$ trend starting at 1.02±0.01 for 100 keV and c at ∼1.00 for the MeV photon energies. In contrast, the Palmer *et al.* composition showed an *increasing* $f^{-1}\left(Q\right)$ trend with energy starting at 0.947±0.006 and plateauing at ∼0.99 for the MeV photon beams. Specifically, $f^{-1}\left(Q\right)$ varied by (2.4±0.3)% from 100 keV photons to 15 MeV photons for the Bekerat et al. composition and by (4.5±0.7)% for the Palmer et al. composition. $f^{-1}\left(Q\right)$ as a function of depth for electrons (Figure 3b,c) followed similar trends for the two film compositions. The high energy electron beams showed a relatively flat response with depth; however, $f^{-1}\left(Q\right)$ for the 5 and 10 MeV beams slightly increased and then fell off dramatically as the dose fell (note that the 5 and 10 MeV range was cut off as the dose was near zero). Finally, $f^{-1}\left(Q\right)$ as a function of depth for photon beams proved to be relatively flat for all energies and both film compositions, except for 150 keV which decreased slightly with depth. All of the kiloelectronvolt photon beams showed film under‐responses for both compositions, except for 100 keV photons with the Bekerat et al. composition which had an average over‐response of (2.2±0.7)% from 5 to 50 mm.

## 4 DISCUSSION

This study investigates the absorbed‐dose energy dependence of two EBT3 and EBT4 compositions, as defined in Table 1, for 5, 10, 50, 100, and 200 MeV electron beams and 100, 150, 1, 2.5, 5, 10, 12.5, and 15 MeV photon beams through TOPAS MC simulations.

The electron depth‐dose curves in Figure 2a behaved as expected. The 5 and 10 MeV depth doses decreased to zero or minimal dose at depths shallower than 50 mm and the high energy (≥ 50 MeV) electrons maintained relatively flat depth‐dose profiles throughout the 50 mm water phantom. Considering the clinical electron energies, the (0.7±0.2)% and (1.1±0.2)% variation between 5 and 10 MeV electrons found in this work for the Bekerat et al. and Palmer et al. compositions proved consistent with a similar study on absorbed‐dose energy response by Sipila et al. which found a (0.5±1.6)% variation in $f^{-1}\left(Q\right)$ for 6 to 16 MeV electron beams.^26^ However, the discrepancy between film and water increased significantly with depth for these clinical electron energies. This can be attributed to the higher attenuation of electron beams in film compared to water, which introduces additional depth dose measurement error when a stack of film is used in a single experiment. In additional 5 MeV simulations to confirm that the large drop in $f^{-1}\left(Q\right)$ was a result of film stacking, the difference in $f^{-1}\left(Q\right)$ between 5 and 20 mm depth was within 0.7% for both film compositions when only one film was simulated at a time. The higher energy electrons (≥ 50 MeV), presented film under‐responses on the order of 2%–3% consistently with depth, significantly larger than in the clinical electron beams. However, a 50 MeV beam is already outside of the 100 keV to 18 MeV energy range recommended by the manufacturer.^7^ With proper calibration, these films could likely be used for such high‐energy electron beams. In fact, EBT3 GAFChromic films have been and continue to be used on the CERN Linear Electron Accelerator for Research (CLEAR, 30–220 MeV).^19^, ^20^, ^21^ However, in these studies, films were calibrated with 5.5, 20, and 21 MeV electrons, which based on this study, could yield dose measurement errors up to (2.5±0.2)% due to the greater extrinsic film response of 5 MeV electrons compared to 200 MeV electrons.

For megaelectronvolt photons, the Bekerat et al. composition showed minimal extrinsic independence with deviations of less than 1.0% from unity for all megaelectronvolt energies and film depths. The Palmer et al. composition resulted in an average under‐response of (1.1±0.5)% for all megaelectronvolt photon beams and film depths. At 150 keV, $f^{-1}\left(Q\right)$ for the Bekerat et al. composition was closer to unity than the more recent Palmer et al. composition, similar to the electron and megaelectronvolt photon beams. Interestingly, at 100 keV, the Bekerat et al. composition resulted in a (2.2±0.7)% film over‐response on average across all films. In contrast, the Palmer et al. composition resulted in a (5.2±0.7)% under‐response on average across the films. The (4.5±0.7)% and (2.4±0.3)% variation in $f^{-1}\left(Q\right)$ for photons from 100 keV to 15 MeV for the Palmer et al. and Bekerat et al. compositions, respectively, indicates the importance of calibrating films with the energy intended for measurement— primarily in the case of low‐energy x‐ray measurements such as for the 100 and 150 keV beams investigated in this study. The (2.4±0.3)% variation in $f^{-1}\left(Q\right)$ for the Bekerat et al. composition proved consistent with a study by Hermida‐Lopez et al. which similarly found a 2.3% variation across 10 keV to 18 MeV for the Bekerat et al. film composition.^25^

**FIGURE 4:**
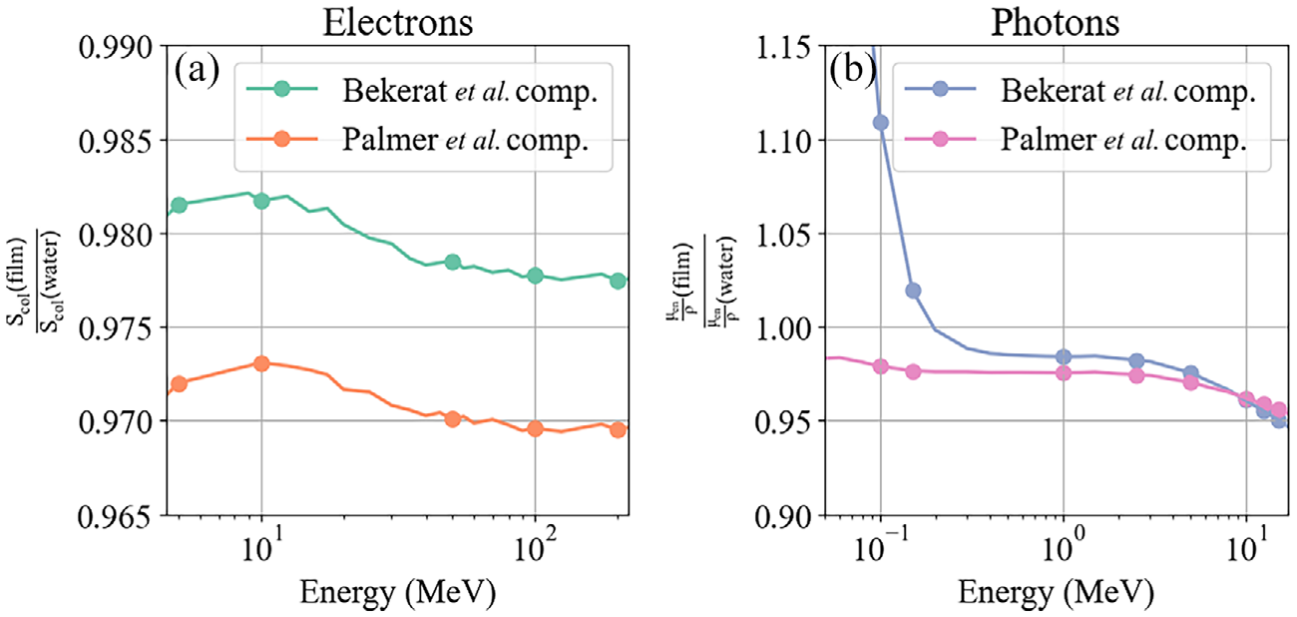
(a) The ratio of active layer electron mass collisional stopping power to that of water as a function of energy. (b) The ratio of the active layer mass energy absorption coefficient to that of water as a function of energy. Data are from the NIST database and markers indicate the specific energies considered in this study.

To further analyze the results, the electron mass collisional stopping power of the active layer of film, $S_{col}\left(film\right)$, was calculated for each composition using the National Institute of Standards and Technology (NIST) Electron STopping powers and Range tables (ESTAR)Too database^36^ and the ratio to the mass collisional stopping power of water, $S_{col}\left(water\right)$, was then calculated as a function of electron energy (see Figure 4a). Since collisional stopping power is related to absorbed‐dose, this ratio should have a similar trend to $f^{-1}\left(Q\right)$ in Figure 3a. It should be noted that $f^{-1}\left(Q\right)$ will likely differ since the ratio of collisional stopping powers will not account for the dose that will arise from Bremsstrahlung photon production. Similarly, for photons, the ratio of mass energy absorption coefficients was calculated for the active layer of film to water, $\frac{\frac{\mu_{en}}{\rho }\left(film\right)}{\frac{\mu_{en}}{\rho }\left(water\right)}$ which should be similar to the results seen for photons for $f^{-1}\left(Q\right)$ in Figure 3a since mass energy absorption coefficients are proportional to absorbed‐dose.

The electron film to water absorbed‐dose response, $f^{-1}\left(Q\right)$, did appear to follow a reasonably similar trend with energy to the predicted behavior estimated with mass collisional stopping powers (see Figure 4a). Films showed an under‐response that agreed within 2% of the stopping power ratios and followed a similar trend of better agreement with water at clinical 5–10 MeV electron beam energies and worse agreement at high energies.

The comparison of $f^{-1}\left(Q\right)$ for photons in Figure 3a and the mass energy absorption coefficient ratio $\frac{\frac{\mu_{en}}{\rho }\left(film\right)}{\frac{\mu_{en}}{\rho }\left(water\right)}$ in Figure 4b shows that the mass‐energy absorption coefficients did predict the over‐response of the Bekerat et al. composition at 100 keV but by a much greater degree of 11% compared to 2%. However, the mass‐energy absorption coefficient ratio did not predict the great under‐response of the Palmer et al. composition at 100 keV. For the other photon beam energies, the mass absorption coefficients predicted worse agreement than what was simulated by dose absorption, including a slightly worse response for the higher megaelectronvolt energies that were considered, nevertheless, the mass absorption coefficient ratios were accurate within ∼5% of $f^{-1}\left(Q\right)$, except for 100 keV.

The differences in the absorbed‐dose energy response between the Bekerat et al. and Palmer et al. film compositions can likely be predominantly attributed to the differences in aluminium, sulfur, chlorine, and bromine which were shown to have a significant impact on the absorbed‐dose energy response of EBT3 in the study by Bekerat et al.^32^

Considering the clinical electron energies of this study (5 and 10 MeV), the difference in $f^{-1}\left(Q\right)$ was ≤(1.1±0.2)% for the first film at 5 mm depth for both film compositions. Therefore measurements using a 5 MeV beam and a 10 MeV calibration (or vice versa) should result in ≤(1.1±0.2)% dose difference attributed to the extrinsic energy dependence of the film alone. Similarly, the clinical photon energies of this study (1, 2.5, 5, 10, 12.5, and 15 MeV) had variations in $f^{-1}\left(Q\right)\;\leq \left(1.0\pm 0.4\right)\%$, suggesting the same error in calibrating with a different clinical energy than the one being used for measurements based only on the extrinsic energy dependence. However, it should be noted that monoenergetic sources were used in this study. Monoenergetic electron beams were used as an acceptable first approximation to clinical linac electron beams given their low energy spread (e.g., 0.04 MeV full width at half maximum for the spectrum central peak at the exit window for the Synergy Elekta accelerator^37^). However, it should be noted that clinical linacs produce photon spectra rather than the monoenergetic beams considered in this work. Monoenergetic sources were used to extrapolate the film response to photons of a particular energy, similar to previous works.^23^, ^25^ The results of this study clearly show that lower‐energy x‐rays can have significantly different $f^{-1}\left(Q\right)$ values, reaffirming the importance of calibrating films with the beam that will be used for measurement when low‐energy x‐rays are present, particularly for x‐ray tube sources.

Finally, it is important to note that this work considers the absorbed‐dose energy dependence, f(Q), which does not account for the intrinsic energy dependence of film dosimetry. Intrinsic energy dependence accounts for the polymerization efficiency in the active layer resulting in color change and can impact the overall energy dependence.

## 5 CONCLUSIONS

The absorbed‐dose‐response of EBT3 and EBT4 films was calculated using MC and the effects of film composition, beam energy, and particle type on dose‐to‐film compared to dose‐to‐water were examined. Two known EBT3 compositions were simulated and given that the atomic composition and geometry of EBT4 films are the same as EBT3 films, the results for the more recent Palmer et al. composition should extend to EBT4. Differences up to (7.3±0.8)% in the absorbed‐dose response were seen between the two film compositions considered in this study. This work also found that stacking films for depth dose measurements for 5 to 10 MeV energy electron beams can lead to large dose discrepancies of up to (10.2±1.1)% at depth. Most importantly, calibration with a different energy beam could lead to measurement errors of up to (4.5±0.7)% considering photons ranging from 100 keV to 15 MeV and up to (2.5±0.2)% for electrons from 5 to 200 MeV. For example, film measurements using the final EBT3 composition, which is the current EBT4 composition, performed at 200 MeV using film calibrated with a 5 MeV clinical electron beam could result in a ∼2.5% lower dose measurement simply due to the absorbed‐dose energy dependence.

## AUTHOR CONTRIBUTIONS

*Conception and design*: Nathan Clements and Magdalena Bazalova‐Carter. *Data collection*: Nathan Clements. *Data analysis and manuscript writing*: Nathan Clements. *Edits and final approval of manuscript*: Nathan Clements and Magdalena Bazalova‐Carter.

## CONFLICT OF INTEREST STATEMENT

The authors declare no conflicts of interest.

## Footnotes

1 Personal correspondence with Ashland Inc.
